# Interpersonal Physiological Synchrony Predicts Group Cohesion

**DOI:** 10.3389/fnhum.2022.903407

**Published:** 2022-07-12

**Authors:** Alon Tomashin, Ilanit Gordon, Sebastian Wallot

**Affiliations:** ^1^Department of Psychology, Bar-Ilan University, Ramat Gan, Israel; ^2^The Gonda Multidisciplinary Brain Research Center, Bar-Ilan University, Ramat Gan, Israel; ^3^Max Planck Institute for Empirical Aesthetics, Frankfurt, Germany; ^4^Department of Psychology, Leuphana University of Lüneburg, Lüneburg, Germany

**Keywords:** physiological synchrony, cohesion, group-level synchrony, individual-level-synchrony, recurrence quantification analysis

## Abstract

A key emergent property of group social dynamic is synchrony–the coordination of actions, emotions, or physiological processes between group members. Despite this fact and the inherent nested structure of groups, little research has assessed physiological synchronization between group members from a multi-level perspective, thus limiting a full understanding of the dynamics between members. To address this gap of knowledge we re-analyzed a large dataset (*N* = 261) comprising physiological and psychological data that were collected in two laboratory studies that involved two different social group tasks. In both studies, following the group task, members reported their experience of group cohesion via questionnaires. We utilized a non-linear analysis method-multidimensional recurrence quantification analysis that allowed us to represent physiological synchronization in cardiological interbeat intervals between group members at the individual-level and at the group-level. We found that across studies and their conditions, the change in physiological synchrony from baseline to group interaction predicted a psychological sense of group cohesion. This result was evident both at the individual and the group levels and was not modified by the context of the interaction. The individual- and group-level effects were highly correlated. These results indicate that the relationship between synchrony and cohesion is a multilayered construct. We re-affirm the role of physiological synchrony for cohesion in groups. Future studies are needed to crystallize our understanding of the differences and similarities between synchrony at the individual-level and synchrony at the group level to illuminate under which conditions one of these levels has primacy, or how they interact.

## Introduction

Social interactions entail the coordination of different biobehavioral processes between individuals, and a prominent pattern of coordination that has been increasingly researched in the past decades is that of interpersonal synchrony. Interpersonal synchrony is defined as “the spontaneous rhythmic and temporal coordination of actions, emotions, thoughts and physiological processes” between individuals (Mayo and Gordon, 2020, p. 1) which is a meaningful aspect of various categories of human interactions. Initiated early on developmentally, biological and behavioral synchrony during parent-infant interactions is considered one of the building blocks of attachment and social development (Feldman, 2007). Moreover, coupling in language, emotion, movement, and autonomic physiological processes is manifested both in close relationships but also during first-time interactions between strangers (Koole and Tschacher, 2016; Azhari et al., 2020).

Synchrony in different modalities is considered to be dependent on a common neural mechanism pertaining to social herding and has been related to several prosocial outcomes (Kokal et al., 2011; Rennung and Göritz, 2016; Shamay-Tsoory et al., 2019; Gordon et al., 2020a). Among these, cohesion has been a major topic in group studies due to its contribution to the group’s performance, connectivity, and effectiveness (Evans and Dion, 1991; Burlingame et al., 2001; Beal et al., 2003). Furthermore, group cohesion was found to be vastly connected to coordinated behavior, whilst there is sparse evidence regarding its association with physiological synchrony (Conradt and Roper, 2000; Wiltermuth and Heath, 2009; Valdesolo et al., 2010; Jackson et al., 2018).

A large body of research focused on physiological synchrony, the temporal coordination in physiological processes between two individuals or more, and how it is linked to relationship characteristics or social outcome (Palumbo et al., 2017; Mayo et al., 2021). Due to their high accessibility, cardiovascular measures are widely used in interpersonal synchrony research. Specifically, coupling in heart rate (HR) or cardiological interbeat intervals (IBIs) were found to be positively associated with beneficial relationship elements, although there is heterogeneity in reported results regarding the effects of this type of physiological synchronization (Järvelä et al., 2013, 2016; Prochazkova et al., 2019; Mayo et al., 2021).

In the present study, we focus on assessing physiological synchronization by calculating the continuous covariation of cardiological IBIs between group members. IBI represents the time between consecutive heartbeats, and it is regulated dynamically by both branches of the autonomic nervous system (ANS): The parasympathetic branch and the sympathetic branch. Synchrony in IBIs has been shown to support social bonding between mother and infant (Feldman et al., 2011), and has also been shown to emerge as a consequence of shared psychological states (Golland et al., 2015, 2019) or active cooperation (Mønster et al., 2016) between adults. In a previous study, we found IBI synchrony was related to a sense of group cohesion and predicted behavioral coordination in group members (Gordon et al., 2020b). A recent meta-analysis of the outcomes of physiological synchrony (Mayo et al., 2021) pointed to an overall positive effect of IBI synchrony on group outcomes such as cohesion, commitment, and performance.

Notwithstanding, social synchrony dynamics are complex and there are continuous shifts in and out of synchrony throughout social interactions. The flow of social interactions relies on a flexible system allowing a transition between synchronous and asynchronous interactions patterns (Mayo and Gordon, 2020). As such, context is an important determinant of the meaning of physiological synchrony (Danyluck and Page-Gould, 2019) since it poses different demands on the balance between the need for synchronization and the need for segregation (Mayo and Gordon, 2020). Will synchrony during an argument indeed lead to a sense of cohesion between partners, or should brainstorming involve more synchrony between partners? These questions highlight how context adds another layer of complexity to physiological coupling as different environments and different tasks yield different correlations between physiological synchrony and relationship outcomes (Mayo et al., 2021). For instance, Danyluck and Page-Gould (2019) found that both verbal communication and social framework changed the meaning of physiological synchrony (in parasympathetic nervous system activity)–specifically, its relationship with friendship interest.

While there has been much progress in terms of uncovering coordination patterns in various domains, as we have summarized above, several questions remain unanswered. Among these are questions pertaining to how physiological synchrony is shaped by contextual aspects of the situation and task, and at what level in the group such coordination is organized. Regarding the latter aspect, a key challenge remains when conceptualizing the interaction of two or more individuals. Is it mainly an interaction of otherwise separate individuals, or do these individuals behave more like a superorganism (Emerson, 1939)? The individualistic (or intra-personal) account has probably been the most clearly spelled-out by investigations of linguistic alignment during conversations (Pickering and Garrod, 2004, 2013), where a specific linguistic mechanism is proposed that controls the interaction of two otherwise separate actors (Fusaroli and Tylén, 2016). On the other hand, dyads or groups that interact have been proposed to function in terms of synergetic relationships (Fusaroli et al., 2014), where the individual actors are bound together by their interactions in a more intricate way, so to act as a superorganism.

For dyadic interactions, this distinction is more of a conceptual nature, as analyzing synchrony in such interactions from the perspective of two separate but interacting entities vs. a synergetic whole is relatively immaterial when it comes to the concrete analysis approach taken. However, multi-level consideration becomes tangible and pertinent when addressing the behavior of groups bigger than two. The complication of investigating coupling in groups is related to the fact that interaction dynamics (Arrow et al., 2000; Moreland, 2010; Williams, 2010; Jones, 2014; Kenny and Kashy, 2014), for example in terms of synchrony, can in principle be situated at the level of the individual, among certain or all dyads of that group, or at higher group levels–the latter would strongly favor the interpretation of group coordination as synergetic (Riley et al., 2011; Wallot et al., 2016b).

Hence, when studying relationships among triads, it is favorable to be attentive to various levels of synchrony within the group–individual tendency to synchronize, as well as dyadic and triadic connections (Gordon et al., 2021). With sparse literature regarding physiological synchrony in groups of more than two people, the current study focuses on studying the role of cardiovascular coupling in determining triadic relationships during collaborative small-group interactions. Specifically, building on initial results indicating an association between group cohesion and physiological synchrony in HR or IBIs (Mønster et al., 2016; Gordon et al., 2020b), we were interested in exploring how this relationship changes at different levels of synchrony within a group and in various contexts.

The investigation of the contextual factor is important because different contexts have the power to shape interactions in different ways, specifically in groups. While free interaction among a group of people might allow for the emergence of higher group-level dynamics, where the group behaves as a synergetic superorganism, we can imagine that other contexts that heavily restrict interaction among members of the group prevent such kind of coordination. For example, groups of people working at an assembly line act in an environment where each member only provides input to the next member in the line, and the mechanized pace and structure of the interaction likely delimits bidirectional interpersonal coordination, making the group behave as the sum of individual behaviors or the sum its dyadic interactions.

From these considerations follow the concrete aims of the present study. First, we aimed to assess if physiological synchrony in IBI between group members predicts cohesion. Our second aim was to investigate the modulatory role of context on the relationship between synchrony and cohesion by comparing two different experimental social tasks with two conditions each. In the current study, we thus present a re-analysis of data sets from two studies that examined the outcomes of physiological synchrony in groups (See Gordon et al., 2020a,b). The first study involved a social drumming task, and the second study involved a group decision-making task. In both studies, triads of participants interacted under different contextual conditions: In one task, participants drummed together in a structured manner without verbal communication, and in another study, they reached a unanimous group decision in ranking the order of several items that would aid their survival if they were stranded on a deserted island. As mentioned above, previous studies, including the drumming study from our own lab (Miles et al., 2011; Tarr et al., 2016; Gordon et al., 2020b) have shown initial evidence that group cohesion, as measured with a self-report questionnaire after the group interaction phase, was related to interpersonal synchronization.

The investigation of data from two different studies with different tasks is of importance, because they differ in important aspects in their structure: The decision-making task is relatively unstructured, allowing for free social interaction between group members, while the drumming task is more structured and aimed at prompting a more direct, stimulus-driven interaction between group members, which relies more on the individual contribution of group members. We set out to assess if the different task characteristics will lead to a different relationship between physiological synchrony and cohesion.

Our final aim was to assess at which level these effects (the link between physiological synchrony and cohesion) occur–individual- or group-level, or whether both of these levels contribute. Particularly if we were to find a contribution of group-level synchrony to perceived cohesion by group members, this could be taken as evidence for synergetic group interactions, where the group behavior acts as a superorganism of sorts.

In order to investigate group-level processes, we employed multidimensional recurrence quantification analysis (MdRQA) (Wallot et al., 2016b). While many analysis techniques exist that allow computation of synchrony measures for dyads, such as cross-correlation (e.g., Konvalinka et al., 2010), relative-phase analysis (e.g., Lumsden et al., 2012), or cross-recurrence analysis (e.g., Shockley et al., 2003), the simultaneous integration of more than two time series, such as data from triads or even bigger groups is more difficult. However, MdRQA allows to compute measures of coupling and synchrony at different group levels–for dyadic, triadic, or greater groups–and thus makes it possible to look at emergent coordination at the group level. Furthermore, it is possible to use MdRQA to compute measures of individual-level (Gordon et al., 2021), or more precisely, the degree to which individual participants are involved in the synchronous interactions with other group members. Accordingly, MdRQA is well suited to investigate effects at individual, as well as group-level effects of synchrony.

As noted above, based on previous work (Gordon et al., 2021), we expect to see a positive effect of IBI synchrony on cohesion, as this physiological synchrony construct may indicate positive joint arousal during a shared task (Konvalinka et al., 2011) linked to closeness between individuals comprising the group. Whether group cohesion is linked to physiological interactions which are based on local interactions between individuals or are situated on the simultaneous interaction among members at the group level is currently an open question, and most likely also a function of context and task. This question can be tested by comparing synchrony measures representing different group levels, as we will do in the following study by using MdRQA: This analysis allows us to compute group-level interactions, that do not only consider dyadic interactions, but also interactions that emerge among more than two members simultaneously. Finding effects of IBI synchrony at this group-level may be considered as evidence for synergetic group processes, where the group behaves more like a superorganism of sorts (Emerson, 1939), rather than the sum of the individuals (or dyads) comprising a group. Conversely, finding that such effects are located at the lower levels of interaction may indicate that a group is quite well described by assessing individual’s participation in all dyadic interactions.

In line with the above, we sum the aims of the current study: (1) To examine if physiological synchrony in IBIs relates positively to group cohesion in a large dataset across multiple social tasks. (2) To assess at which level do these effects occur–individual or group or an interaction of the two. (3) To understand the meaning of an individual-level vs. group-level effect of physiological synchrony on cohesion.

## Materials and Methods

### Participants and Procedure

This study was a re-analysis of data from a total of 261 participants (72.4% female, mean age = 23.32, SD = 3.1) who participated in one of two experiments (from here on labeled as “drumming” and “decision-making”) conducted in our lab during 2017 and 2018. Most participants were Psychology undergraduate students and were compensated with course credits, while others received payment. The study was approved by Bar Ilan University Department of Psychology Ethics Committee, and every participant provided informed consent.

Both experiments aimed to investigate the relationship between interpersonal coordination of physiological markers and group outcomes such as cohesion and efficacy. Both were conducted with groups of three persons, which were connected to MindWare Mobile Record (MindWare Technologies Ltd., Gahanna, OH, United States) for electrocardiogram monitoring. Out of 101 groups, we analyzed data from 87 triads (“Drumming”–45, “Decision-making”–42) due to incomplete or corrupted physiological data.

**FIGURE 1:**
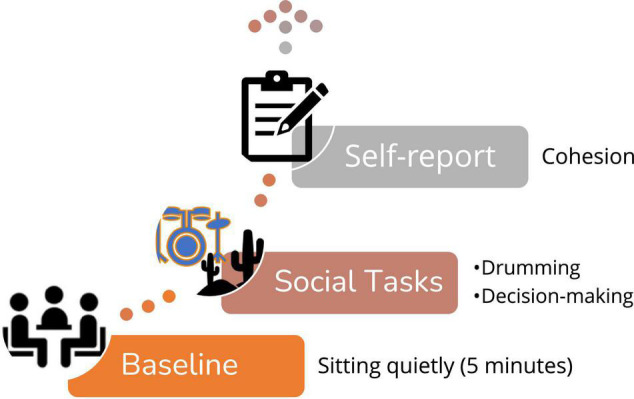
The common phases of the two experiments – “drumming” and “decision making”.

In the current study, we focus on the first two stages of each experiment–a baseline phase and a group social interaction phase (see Figure 1). The baseline phase in both studies entailed participants sitting quietly together, not talking or doing anything for 5 min. They were instructed to either focus on the wall or a certain object in the room or to close their eyes and relax. After the baseline, a social interaction phase commenced. During the “drumming” experiment (Gordon et al., 2020a), participants took part in a drumming task where they were asked to tap on their electronic drumming pad (Roland V-Drum) to a specific tempo that was broadcast in the room via a speaker. During the “decision-making” experiment, participants completed a well-known task in which they ranked, individually and as a group, 15 items based on their relevance to the group’s survival after an airplane crash–a version of the Desert Survival Task (DST) (Lafferty et al., 1974). Both the drumming study and the decision-making study included two task condition. For the drumming task–half of the groups heard a predictable tempo and half of the groups heard a non-predictable tempo. For the decision-making task half of the group were led by a polite experimenter and half were led by an impolite experimenter. In both studies, after the group social interaction, participants filled questionnaires regarding their sense of group cohesion. Our analyses of physiological data are based on cardiological IBIs data that was recorded continuously throughout the experiments from all group members (at baseline and during the interaction) as well as on the self-reported group cohesion score (Podsakoff and MacKenzie, 1995).

### Cohesion Questionnaire

Group cohesion was measured using four items of the widely used questionnaire by Podsakoff and MacKenzie (1995). Each participant responded how well he or she can relate to the following statements on a 1–6 Likert scale: “If possible, I would be happy to participate in another group experiment with the members of my current work group”; “My group worked together as a team”; “We were cooperative with each other”; and “We knew that we could rely on one another” and “We were supportive.” Individual’s experience of group cohesion was calculated as the average of the above scores.

### Collection and Pre-processing of Physiological Data

Electrocardiograms were obtained from group members using a modified lead-II configuration. Respiratory data were derived from the standard tetrapolar electrode procedure for the impedance cardiogram described elsewhere (Sherwood et al., 1990). Electrodes were transmitting synchronously and wirelessly to the control room at a sampling rate of 500 Hz. Electrocardiograms were analyzed in MindWare Technology’s HRV 3.1.4 application and amplified by a gain of 1,000 and filtered with a hamming windowing function. Trained coders in the lab reviewed data manually and visually inspected all data to ensure the removal of artifacts and ectopic beats (Berntson et al., 1997). Inter-beat-intervals (IBI) were extracted from the continuous ECG data. Note that IBI time series differed in length across individuals. Hence, for the purpose of correlating IBI data between members of dyads or groups, we had to trim the longer time series to the shortest time series length for each particular dyad or group. This was done by removing the excess data points at the end of the longer series.

### Calculating Physiological Synchrony

To quantify physiological synchrony, we conducted MdRQA (Wallot et al., 2016b) on the IBI time series using a software implementation in MATLAB version 2021b (The MathWorks, Inc.). This method is suitable for physiological data primarily due to its ability to capture the correlations among multivariate signals. Furthermore, we analyzed our data on both the individual and triadic levels (Gordon et al., 2021).

Similar to other recurrence-based methods (Webber and Zbilut, 1994; Marwan and Kurths, 2002), MdRQA begins with a matrix of distances between pairs of data points in the time series (the IBI data obtained from each participant). For example, if we have three time-series with four data points each *x*_1_ = [1, 1, 2, 25], *x*_2_ = [1, 1, 3, 40] and *x*_3_ = [1, 1, 1, 99], then these three time series provide us with four coordinates in their joint phase-space: *c*1 = [1, 1, 1], *c*2 = [1, 1, 1], *c*3 = [2, 3, 1], and *c*4 = [25, 40, 99]. Table 1 charts the distance matrix between these coordinates.

**TABLE 1:** Distances between the four coordinates.

	c1	c2	c3	c4
c1	0	–	–	–
c2	0	0	–	–
c3	2.2	2.2	0	–
c4	108.2	108.2	107.3	0

**FIGURE 2:**
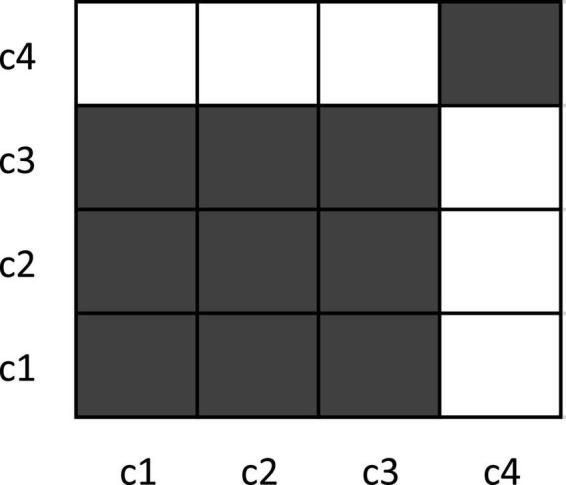
Example recurrence plot for toy data. The dark squares in the plot indicate recurrence (identical/similar coordinates), while the white points indicate the absence of recurrence. Note that the recurrence plot (RP) is, by convention, rotated by 90° compared to the conventional display of a matrix, so that time runs from the lower-left to the upper-right of the plot. Recurrence measures for this plot are: %REC = 62.5%; %LAM = 90%; meanV = 3; maxV = 3. See text for how these values are computed.

If we look at the distances in Table 1, we see that coordinates c1 and c2 are identical–their distance is zero, Hence, the first and second coordinate based on the first and second data point of the three time series are recurrent. The distance to the third data point is bigger than zero–so c1 and c2 are not identical to c3, but similar. Finally, the distance to c4 is comparatively big. c1, c2, and c3 are not very similar to c4. For continuous data that might also include measurement noise as a source of variability, we cannot simply count only identical coordinates as recurrent, but we need to define some range within which two coordinates are counted as recurrent, albeit not being identical. Hence, we determine two points as recurrent (i.e., similar) if their distance is under a preset threshold; otherwise, they would count as non-recurrent (different). Applying such a threshold results in a binary recurrence plot (RP) (Figure 2) of recurrent and non-recurrent data points. If we apply a threshold value of *r* = ±3, the distance matrix in Table 1 yields a recurrence plot as portrayed in Figure 2.

There are multiple RQA outcome measures that evaluate different aspects of the RP (Marwan et al., 2007). The simplest measures is percent recurrence (%REC), which quantifies the raw amount of individual instances (coordinates) that recur with each other. It is the sum of all recurrences divided by the size of the plot. Further measures are percent laminarity (%LAM), which quantifies the degree to which recurrences appear in larger patterns. It is calculated as the sum of all recurrence points having at least a single vertical neighbor divided by the number of all recurrence points. Further, there is the average vertical line length, which captures the average duration of such patterns (meanV), calculated as the average length of vertical lines of recurrences on the plot, and there is the maximum vertical line length (maxV), which captures the longest period over which the time-series form such a pattern, and is captured by the maximum number of vertically adjacent recurrence points. There are further measures (e.g., Marwan et al., 2007).

**FIGURE 3:**
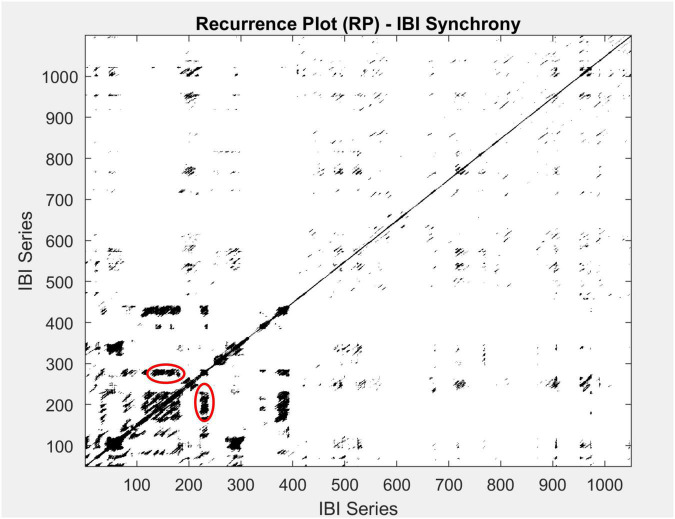
An example of a multidimensional recurrence plot for one group participating in this study. Both the *x*-axis and *y*-axis represent the IBI time series. Blackened areas are recurrent points, and vertical lines are circled in red. To quantify the recurrence rate (REC%), we calculate the percentage of recurrent points across the matrix. Further, to identify recurrence patterns in time, we use LAM%, the rate of recurrence points with a vertical neighbor, and the length of the vertical lines (meanV and maxV).

Here, we focused on recurrence rate (REC%), laminarity (LAM%, percentage of recurrence points with vertical neighbor), and both the maximum and average lengths of the vertical lines (meanV and maxV). We did so, because recurrences of signals that have a substantial stochastic component or a of noise-type show up in terms of squares and patches of recurrences, which are better captured by the vertical lines on a recurrence plot, as can be seen in the plot presented in Figure 3.

To conduct recurrence quantification analysis, further parameters must be set, such as the delay and embedding dimension parameters. These parameters are used to recover higher-order dynamics from the (potentially) lower-order number of time series which have been recorded from a system (Takens, 1981). The delay parameter is estimated by the first local minimum of the average mutual information function of the time series to be subjected to MdRQA. The embedding dimension parameter is estimated by the false-nearest-neighbor function, at the point where this function bottoms out. For a detailed introduction to multivariate recurrent analysis, and the parameter estimation procedure, see Wallot and Leonardi (2018).

For MdRQA, the delay parameter (d), as well as the embedding parameter (m), were approximated using a multivariate mutual information and false-nearest neighbor functions for parameter estimation applied through MATLAB (Wallot and Mønster, 2018). Here, the embedding parameters are estimated for each triad or dyad. Then, these estimates are averaged over the whole sample and rounded to the nearest high integer. Then, these values are used for each data set, which allows to compare data sets with the sample based on the same embedding parameters (see Wallot and Leonardi, 2018). Furthermore, the threshold parameter (r) was fixed across data sets so that each data set entered the analysis with the same value for *r* and the value for r was chosen to provide an average of REC% between 1 and 5% (Webber and Zbilut, 2005) across all data sets by calculating the recurrence rate for a series of optional *r*-values until an appropriate REC% was found.

As stated above, we aimed to capture both individual contributions to the group’s physiological synchrony, as well as the group’s physiological synchrony as a single entity. To pursue these goals, we conducted MdRQA according to guidelines described by Gordon et al. (2021) on two distinct levels–individual and triadic. That is, to investigate group-level dynamics, which contain the interactions between all group members over time (Wallot et al., 2016b), the time-series for all members of the group were subjected simultaneously to MdRQA (as in our toy example above) and recurrence measures were then computed.

In order to capture each individual’s participation in the group dynamics (a so-called individual-level synchrony), we computed all possible dyadic recurrence plots and recurrence measures for every individual in a group and then averaged these for each individual. For instance, participant A’s scores were computed as the average recurrence measures of data from participants A and B (Dyad I) and data from participants A and C (Dyad II). Similarly, participant B’s scores were computed as the average recurrence measures of data from participants B and A (Dyad I) and data from participants B and C (Dyad III), and so forth.

Note that different parameters were assigned for dyads and triads (Table 2), while parameters were kept constant across the set of triads and dyads, respectively. This was done to facilitate the comparability of the MdRQA results across data sets. Furthermore, we generated false-pair surrogates by randomly partnering participants’ time-series with others from different groups (Richardson and Dale, 2005). The same analysis (with the same sets of parameters) was applied on the fabricated groups to indicate random recurrence levels.

**TABLE 2:** Multidimensional recurrence quantification analysis (MdRQA) parameters assigned for dyads and triads.

Parameters	Delay	Embedding dimension	Threshold	Norm
Dyad	2	7	0.457	Euclidean distance norm
Triad	2	7	0.51	Euclidean distance norm

*The IBIs data was not normalized prior to the analysis.*

Due to the strong correlation [Partial and Semi-Partial Correlation R package by Kim (2015)] between the last three parameters (Table 3), we created a primary vertical measure (Vertical Synchrony) which addresses the intermittency in time-series by averaging the z-scores of LAM%, meanV and maxV. While REC% stands for general repetition of values between time series, high vertical synchrony better represents a stronger coupling (Richardson et al., 2008; Wallot et al., 2016a; Proksch et al., 2022).

**TABLE 3:** Correlation matrix, laminarity, mean and maximum vertical line length on the triad level during group interaction.

Partial correlation				

		IBI LAM%	IBI meanV	IBI maxV
IBI LAM%	Pearson’s r	–		
	*p*-value	–		
IBI meanV	Pearson’s r	0.655	–	
	*p*-value	<0.001	–	
IBI maxV	Pearson’s r	0.593	0.915	–
	*p*-value	<0.001	<0.001	–

*Results presented here control for the effects of the experiment (“drumming” and “decision-making” ranked 1 and 2).*

## Results

**FIGURE 4:**
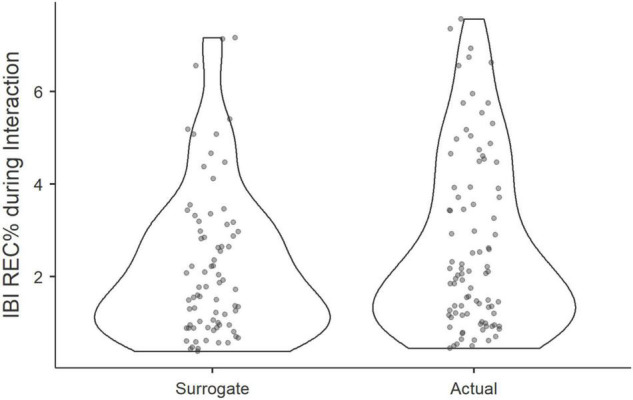
Violin plots show the distribution of REC% of actual and pseudo triads during a group interaction.

First, to test our hypothesis that physiological coupling occurs within groups while interacting, we compared the recurrence rates of IBI, calculated at the triadic level of the MdRQA, to recurrence rates of IBI in false-pair surrogates. Applying Wilcoxon signed-rank test in Jamovi 1.2 (The Jamovi Project, 2021), we discovered that actual groups were more in-sync, as represented by higher REC%, than pseudo-groups (Wilcoxon *T* = 1686, *p* = 0.033), see Figure 4.

**FIGURE 5:**
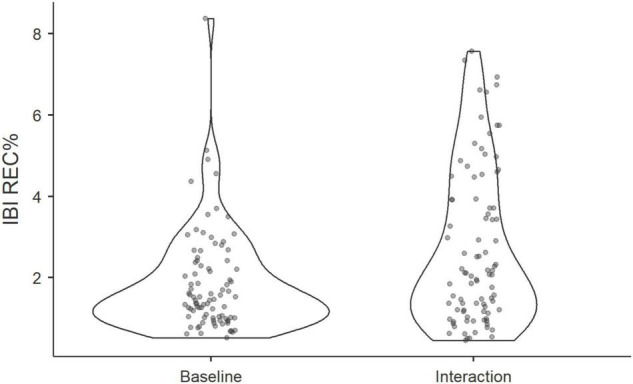
Violin plots show the distribution of REC% of triads at baseline and during the group interaction.

Next, a Wilcoxon rank signed test revealed that the degree of physiological synchrony during the task was higher than the degree of synchrony found at baseline (Wilcoxon *T* = 1018, *p* < 0.001), see Figure 5. However, we did not find significant differences in recurrence rates at baseline compared to those found among surrogate groups (Wilcoxon *T* = 1619, *p* = 0.55). Thus, it appears that IBIs synchrony between group members, which is significantly above chance level, develops when participants act together rather than when they merely sit in the same room together. In so far as IBI-activity captures arousal, it is also plausible that IBI-synchrony is observed in the mere presence of others, without tangible joint activities–such as in a baseline phase where all participants are present in the same room and can see each other, but do not interact in a specific task. However, the results indicate that synchrony during baseline was not significantly higher than what would be expected due to the chance-level of false-pairs.

To test whether synchrony in IBI during social interaction predicts group cohesion, we applied a mixed model in Jamovi using GAMLj module (Gallucci, 2019). As independent predictors, we deployed a combined recurrence measure (Vertical Sync, see Section “Materials and Methods”), and condition while the group (drumming or decision making) acted as a random factor (Table 4). No significant results for cohesion were found for the groups’ synchrony during the task stage of the experiment; in contrast, the effect of baseline synchrony on cohesion was negative (Table 5). Hence, refining the on-task synchrony measure, we created a delta measure (ΔSync) by subtracting the baseline levels of synchrony from the interaction levels.

**TABLE 4:** A mixed model predicting cohesion from IBI synchrony at the interaction.

Group Level IBI sync at interaction model–Fixed effect on cohesion				

	*F*	Numerator df	Denominator df	*p*
Condition	3.12	3	2	0.252
IBI Sync at Interaction	1.75	1	85	0.190
				

**TABLE 5:** A mixed model predicting cohesion from IBI synchrony at baseline.

Group Level IBI sync at baseline model–Fixed effect on cohesion				

	*F*	Numerator df	Denominator df	*p*
Condition	3.31	3	2	0.240
IBI Sync at Baseline	4.38	1	83	0.039

**FIGURE 6:**
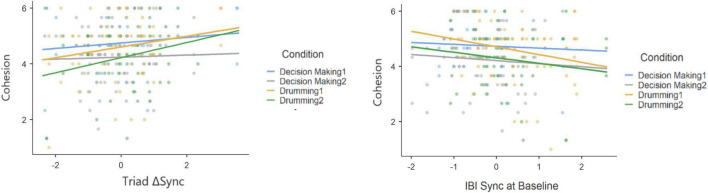
Scatter plots and slopes for the IBI data during task minus baseline (triadic ΔSync) and the baseline data (IBI Sync at Baseline).

While most studies investigating interpersonal synchrony analyzed the degree of synchrony during social interaction, it has also been proposed that a physiological coupling occurs among individuals who share the same space without engaging in an interaction (Golland et al., 2015). The mentioned mere co-presence setting resembles the baseline stage in our experiments in which participants were asked to sit quietly in the same room. Therefore, to account for the outcomes of the collaborative task, we investigated the formation of synchrony by subtracting each group’s degree of synchrony at baseline from its interaction synchrony score. This measure, ΔSync, constitutes the change in joint physiological activity from the first inactive 5 min (i.e., the baseline) to interaction during the shared task. Figure 6 shows the data at baseline in comparison to the interaction minus baseline model.

**FIGURE 7:**
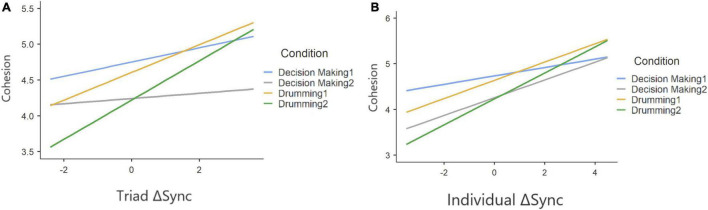
Two-way interaction plot, based on a random slopes and random intercept mixed model, predicting cohesion from ΔSync on triad level **(A)** and individual level **(B)** and on under different conditions (plotted as different colors). The positive correlations indicate that a positive change in IBI synchrony, from baseline to engagement in a group activity, predicts a stronger sense of cohesion among group members.

Our findings indicate that ΔSync was positively related to a participants’ sense of group cohesion when synchrony was calculated at the group level (Table 6) and that experimental condition was associated with cohesion. To test the robustness of this relationship between physiological synchrony and cohesion, we ran a second mixed model predicting cohesion from ΔSync calculated separately for each participant, holding group as a random coefficient (Table 7). Similarly, both experimental condition and individual ΔSync yielded a significant effect on group cohesion. Interestingly, the positive relationship between ΔSync and cohesion was consistent across experimental conditions in both experiments as no interaction effect was found between ΔSync and condition (Figure 7A and Figure 7B).

**TABLE 6:** A mixed model (see equation above) predicting participant’s cohesion from ΔSync (**γ_10_**), task condition (**γ_20_**), and their interaction (**γ_12_**) at the triad level.

Group level ΔSync model–Fixed effect on cohesion				

	*F*	Numerator df	Denominator df	*p*
Condition	3.443	3	114.7	0.019
Triad ΔSync	5.204	1	79.0	0.025
Condition × Triad ΔSync	0.581	3	79.0	0.629

*Group cohesion serves as a random effect (**u**_**0j**_) and is added to the general cohesion average (γ_00_). Marginal R^2^ = 0.074.*

**TABLE 7:** A mixed model predicting cohesion from ΔSync, task condition and their interaction at an individual level.

Individual level ΔSync model–Fixed effect on cohesion				

	*F*	Numerator df	Denominator df	*p*
Condition	3.385	3	80.9	0.022
Triad ΔSync	8.557	1	113.0	0.004
Condition × Triad ΔSync	0.431	3	112.4	0.731

*Marginal R^2^ = 0.08.*

However, model comparisons between a model containing only the individual-level predictors and a model containing both, the individual-level and the group-level predictors suggested that the triadic level of ΔSync does not contribute to the prediction of cohesion beyond the individual-level effects (*X*^2^ = 0.0014, *p* = 0.97).


$$C\,o\,\text{hesio}\,n_{\text{ij}}=\gamma_{00}+u_{0\,j}+\gamma_{10}\cdot \Delta \,S\,y\,n\,c\,+\gamma_{20}\cdot C\,o\,n\,d\,i\,t\,i\,o\,n+\gamma_{12}\cdot I\,n\,t\,\left(\Delta \,S\,y\,n\,c*C\,o\,n\,d\,i\,t\,i\,o\,n\right)+\varepsilon_{\text{ij}}$$

## Discussion

In the present study, we analyzed group interaction data from two different studies–one from a joint drumming and the second from a joint decision-making task. Specifically, we examined how synchronous IBI dynamics at the individual- and triadic-level were related to self-reported group cohesion. Across all conditions, we found a positive effect of physiological synchrony on cohesion. This effect was observed at the individual-level and at the triadic-level. While the reported level of cohesion differed between different study conditions, the effect of IBI synchrony on cohesion was stable across conditions, as no significant interaction between synchrony and condition emerged.

Considering the role of synchronization in social tasks, our results highlight the centrality of physiological synchronization mechanisms as potentially facilitating the formation of groups and their members’ sense of group cohesion. These results are pivotal as experiencing the group as cohesive has been demonstrated to have a strong effect on the group’s objective performance outcomes (Evans and Dion, 1991; Beal et al., 2003). These findings also provide a much called for extension of previous work on the prosocial implications of synchrony from the dyad-level to the much less examined group context (Bernieri and Rosenthal, 1991; Hove and Risen, 2009). More specifically, we provide further evidence for the role of physiological synchrony in IBIs for prosocial effects in groups (Palumbo et al., 2017; Mayo et al., 2021) while utilizing a non-linear analytical approach on two datasets that involve two different types of social tasks–one verbal and the other rhythmic-motor, one very structured and the other only relatively unstructured.

Regarding the question of emergent synchronization at the group-level (i.e., the group-as-superorganism), we found a significant synchronization beyond what would be expected at chance-level. This suggests that IBI synchronization in the groups across the different conditions indeed represents a group behaving to some extent as a superorganism, and that such group-level coordination needs to be taken into account when modeling data in groups bigger than two. However, effects on the individual-level (i.e., how much each member of the group synchronized with the other two members), occurred as well. On the one hand, these results seem to point to multilevel synchronization emerging in the groups, suggesting that the analysis of both, the individual- and the group-level effects of synchrony illuminates a potential multilayered aspect of cohesion as a group phenomenon. As cohesion is evaluated through self-report questionnaires at the individual-level (Evans and Jarvis, 1980; Dion, 2000; Salas et al., 2015), it is intriguing that both individual-level and group-level physiological synchrony contributed to predicting cohesion. While group-level synchrony may reflect the group’s unification and cooperation, the individual-level synchrony emphasizes one’s attraction to the group members and his\her willingness to continue collaborating with the other members. These dual aspects that impact cohesion are important to consider in future research.

However, the results of model comparisons indicated that the group-level dynamics did not substantially contribute to model fit of cohesion values beyond what could be inferred from the individual-level predictors alone. Obviously, in the current data-sets, both sets of predictors where highly correlated. This did not allow us to tell specific sources of contribution on these different levels apart. Accordingly, the results do not yield substantial evidence for emergent coordination among the group members regarding the superorganism hypothesis. However, these results illustrate the importance of measuring group dynamics and coordination on different levels, because sometimes such effects are only present on some or none of the levels (Wallot et al., 2016a; Gordon et al., 2021). Moreover, pitting these effects against each other allows for a more accurate test of the importance of the different levels. If we had only considered group-level dynamics and tested them against surrogate data as well as in terms of their predictive value for cohesion ratings, we might have taken these results as strong evidence of emergent group-level synchronization, while the comparison to the individual-level data calls such a strong conclusion into question.

Our study lays out an innovative perspective on the development of synchrony as a key factor in understanding social group dynamics. By investigating the change in the degrees of synchrony (ΔSync), rather than its absolute value during social interaction, we put more weight on the initial stages of the formation of social bonds. A similar point of view was presented regarding cohesion (Marks et al., 2001), defining it as an emergent state, a dynamic temporal component, that is altered with team experiences. Still, our approach might be more suitable to investigate in newly formed groups of relative strangers that, according to our findings, do not tend to synchronize with each other at baseline prior to the interaction.

It is interesting to note here that in the original drumming study, when we utilized a linear approach to quantifying synchrony during the group task (Gordon et al., 2020a), we found a relationship with cohesion without considering the baseline period. Perhaps this fact has to do with the methodology, in which the linear cross-correlation function as we used it, considered only the strongest correlation close to lag0 to assess synchrony. Conversely, MdRQA looked only at strict lag0 recurrence, but incorporated autocorrelation (i.e., auto-recurrence) information from other lags as well (Marwan et al., 2007). Another explanation for the “baseline” effect, is that the antecedents of meaningful synchronization between group members already exists in the very initial stages of social grouping–what may be termed as a “first impression” effect. This explanation is intriguing but requires further examination in future studies.

It should be noted that we did not find any differences regarding the effect of physiological synchronization on cohesion across tasks or experimental conditions. Even though the two tasks appear to differ in terms of how they implement group coordination, they did not moderate effects of synchrony on cohesion. On the one hand, this suggests that the effects of IBI synchrony on cohesion are very stable across a certain range of tasks and interaction types. On the other hand, it remains unclear what exactly drives differential effects of synchrony in different group settings (Palumbo et al., 2017; Mayo et al., 2021). Future studies should test if negative contexts or competitive ones would yield similar results to the ones we found here.

This is also one of the limitations of the present analyses: The two studies seem to differ in their tasks demands, but they were not specifically designed to manipulate factors that may change the role or meaning of synchronization during interaction (Danyluck and Page-Gould, 2019). Further limitations are a lack of control for gender as well as a homogenous sample mainly consisting of undergraduate students. As a result, we could not account for background differences and in-group or out-group effects, which carry relevance for synchrony’s prosocial effects (Tunçgenç and Cohen, 2016; Cirelli, 2018).

To expand the knowledge on the role of physiological synchrony for group processes, future studies should aim to implement experimental manipulations that are likely to change the meaning or role of synchronization for the group (from a positive to a negative context for instance or from cooperation to competition). Moreover, additional longitudinal research should trail newly formed groups’ development and bond formation alongside the development of physiological synchrony over time. Nonetheless, our results represent an important step in reaching a more crystallized understanding of group processes via objective non-biased measurements of the dynamics of group interactions. We further emphasize the importance of incorporating multilevel representations of synchrony within groups when analyzing the effects of synchronization on cohesion.

## Data Availability Statement

The original contributions presented in this study are included in the article/supplementary material, further inquiries can be directed to the corresponding author.

## Ethics Statement

The study was approved by Bar-Ilan University Department of Psychology Ethics Committee. The patients/participants provided their written informed consent to participate in this study.

## Author Contributions

AT analyzed the data. All authors discussed and interpreted the results and wrote the manuscript.

## Conflict of Interest

The authors declare that the research was conducted in the absence of any commercial or financial relationships that could be construed as a potential conflict of interest.

## Publisher’s Note

All claims expressed in this article are solely those of the authors and do not necessarily represent those of their affiliated organizations, or those of the publisher, the editors and the reviewers. Any product that may be evaluated in this article, or claim that may be made by its manufacturer, is not guaranteed or endorsed by the publisher.
